# Advances in Transcriptomics in the Response to Stress in Plants

**DOI:** 10.1055/s-0040-1714414

**Published:** 2020-08-20

**Authors:** Xiaojuan Wang, Na Li, Wei Li, Xinlei Gao, Muha Cha, Lijin Qin, Lihong Liu

**Affiliations:** 1 ChiFeng University, Hongshan, Chifeng, Inner Mongolia, People’s Republic of China; 2 Inner Mongolia Academy of Forestry Sciences, Inner Mongolia, Saihan, People's Republic of China

**Keywords:** transcriptomics, biotic stress, abiotic stresses

## Abstract

Adverse stress influences the normal growth and development of plants. With the development of molecular biology technology, understanding the molecular mechanism of plants in response to adverse stress has gradually become an important topic for academic exploration. The expression of the transcriptome is dynamic, which reflects the level of expression of all genes in a particular cell, tissue, or organ of an individual organism at a particular stage of growth and development. Transcriptomics can disclose the expression at the whole genome level under stress from the whole transcriptional level, which can be useful in understanding the complex regulatory network associated with the adaptability and tolerance of plants to stress. In this article, we review the application of transcriptomics in understanding the response of plants to biotic stresses such as diseases and insect infestation and abiotic stresses such as water, temperature, salt, and heavy metals to provide a guideline for related research.

## Introduction

Plants in the natural environment often encounter some adverse environmental factors that affect their survival and growth, which are collectively known as adversity. Plant stress generally includes abiotic stress and biotic stress. Abiotic stress is primarily caused due to physical or chemical conditions such as high temperature, drought, chilling injury, high salt content, heavy metals, and mechanical damage. Biotic stress is primarily caused due to various biological factors such as fungi, bacteria, viruses, nematodes, and parasitic plants. To adapt to adversity, plants get adjusted over time at cellular, organ, physiological, biochemical, and molecular levels, which are finally reflected as morphological changes. This adaptation to take precautionary measures is beneficial for the plants to reduce the damage caused by stress. Due to the worsening of the global environment, plant stress has become an important factor restricting the development of modern agriculture, and research on the plant stress response mechanism is also on the increase.

Plant response to stress is a highly complex life process whose molecular mechanism has not yet been fully elucidated. The transcriptome refers to the sum of all RNAs transcribed by a specific cell or tissue in a certain functional state, including mRNA and noncoding RNA. The term transcriptomics refers to the study of gene transcription (type, structure, and function) and regulation rules in cells from the overall level, including noncoding region function study, transcript structure study, gene transcription level study, and new transcriptional region study. Transcriptomics can be used to quantitatively analyze the changes in plant gene expression at a specific time point and in a specific state, reveal the complex regulatory network and expression at the whole genome level under stress, and screen out new genes related to plant resistance. 1 2 With the development of sequencing technology, transcriptome sequencing has been widely used in *Arabidopsis thaliana* , maize, wheat, rice, soybean, and other crops. In this study, our aim was to review the recent advances in transcriptome studies of plant stress, introduce the changes in the differentially expressed genes (DEGs) in the metabolic pathways under plant stress, and look forward to future research trends to provide a guideline for further research on plant stress.

## Transcriptomics in Response to Biotic Stress in Plants

Biological factors have a significant influence on the growth and development of plants. Pathogen infestation and animal feeding can affect plant growth, yield, and quality. Plants can adjust gene expression and enzyme activity in vivo to achieve signal induction and transmission in order to realize the biological effects. Understanding the relationship between the plant transcriptome and biotic stress is an important strategy to investigate stress resistance.

### Advances in Insect Resistance Studies

Every plant is susceptible to attack by other organisms such as insects and pathogens. Plants require a broad range of defense mechanisms to effectively combat invasion by microbial pathogens or attack by herbivorous insects. The signaling molecules SA, JA, and ET have been implicated in several plant–pathogen and plant–insect interactions. The kinetics of SA, JA, and ET produced by *A. thaliana* after being attacked by microbial pathogens and herbivores differ significantly in quantity and time. There is also an overexpression of stress-related genes. By comparing the transcriptional profiles, it has been found that there may be considerable overlap between pathogens and insect-induced mutations of different attack patterns. 3 The transcriptional response of the cotton plant to whitefly infestation involves genes encoding protein kinases, transcription factors, metabolite synthesis, and phytohormone signaling. WRKY40 and transport protein are hub genes that regulate cotton defense against whitefly infestation. It has been reported that silencing GhMPK3 by virus-induced gene silencing (VIGS) resulted in the suppression of the MPK-WRKY-JA and ET pathways and led to enhanced susceptibility to whitefly infestation. 4 Another research showed that expression of the transcription factor AtMYB12 of *A. thaliana* in tobacco resulted in an enhanced expression of the phenylpropanoid pathway-related gene and an increase in flavonol accumulation. AtMYB12 regulates several pathways, particularly the biosynthesis of flavonols. The tobacco transgenic lines developed resistance to the pests *Spodoptera litura* and *Helicoverpa armigera* through the increased accumulation of rutin. Artificial microRNA inhibited the synthesis of flavonoids, resulting in the inhibition of the resistance of AtMYB12 tobacco plants to insects. 5

### Advances in Disease Resistance Research

Pumpkin has a complex PM resistance regulatory network that may involve hormonal signaling pathways, transcription factors, and defense responses. The expression profiles of 16 selected genes from pumpkins affected by powdery mildew demonstrated six differential gene transcription levels in these genes, including bHLH87 (basic helix-loop-helix transcription factor), WRKY21 (WRKY domain), ERF014 (ethylene response factor), HSF (heat stress transcription factor A), MLO3 (mildew locus O), and SGT1 (suppressor of G-two allele of Skp1). These genes were found to be significantly upregulated or downregulated in varieties resistant to powdery mildew. 6 Genes involved in the resistance of *wheat– Thinopyrum* to powdery mildew have also been identified. Of the 39 single genes, 12 were found to be upregulated by 3 to 45 times in the variety SN6306. These single genes primarily encoded kinases, proteases, synthases, and signal transduction proteins, which may play an important role in combating powdery mildew. 7 Triplicate deep transcriptome surveys of leaves from the same wheat line inoculated with powdery mildew (Bgt) and stripe rust (Pst) have also been conducted. Comparison of transcriptional differences and overlaps between Bgt- and Pst-induced stresses showed that the disease-resistant wheat cell lines used a variety of defense mechanisms to enhance their disease resistance, and the expression patterns of genes related to the same defense varied in response to different pathogen infections. The wheat line and the pathogen share certain microbial genetic materials, which can be used to resist infection. 8 The *TIFY* gene family is involved in the process of plant development and their responses to biotic and abiotic stresses. The *VvTIFY9* protein encoding a conserved motif was identified in the grape ( *Vitis vinifera* ). *VvTIFY9* is induced by salicylic acid (SA) and methyl jasmonate (MeJA) and also responds rapidly to *Erysiphe necator* infection in grapes. The defense-related genes *AtPR1* and *AtPDF1.2* are upregulated in overexpressing lines, *Arabidopsis* overexpressing *VvTIFY9* is more resistant to cyclosporine, and *VvTIFY9* is also closely associated with resistance to grape powdery mildew. 9

## Transcriptomics in Response to Abiotic Stress in Plants

A comparative analysis of transcriptome data could be helpful in understanding how plants balance growth and survival under abiotic stress conditions. Drought stress, extreme temperature, salinity, metal ion toxicity, and other abiotic stresses can induce plants to regulate their own physiological, biochemical, molecular, and cellular processes, actively modify the transcriptome, activate stress tolerance mechanisms, or regulate their biological processes to adapt to adverse living conditions. The mechanism of plants to adapt to injury and stress at the transcriptional level can be better understood by analyzing and screening the differentially expressed functional genes in plant tissues and organs under different stress sources and stress intensity, and determining the relationship between key functional genes and resistance.

### Drought Stress

The increasing trend of global warming has subjected plants to widespread drought stress, a phenomenon that has a severe effect on their survival, growth, and distribution. Transcriptome sequencing is used to analyze the expression of the whole genome under drought stress, which is helpful in understanding the related regulatory mechanism of plant adaptation to drought, or improving drought resistance, and in elucidating the mechanism of plant drought response. A total of 11,359 DEGs were identified in sweet potato after PEG6000 treatment, among which 7666 were upregulated and 3693 were downregulated. Abscisic acid (ABA), ethylene (ETH), and jasmonic acid (JA) signaling pathways play a major role in the drought tolerance of sweet potato, 10 wherein two screened proteins, ABI-like protein and Ca ^2+^ -ATPase, were identified as the major components of drought resistance. 11 Whole genome transcriptome analysis of the epidermal cell layer under drought stress in the annual wild barley in Tibet revealed the presence of a unique cross-talk between plant hormone pathways, cell signaling, and membrane transport. Brassinosteroids can participate in the common regulation of stomatal movement along with abscisic acid. 12 Transcriptome analysis was performed on two drought-tolerant genotypes of *Cicer arietinum* L. under drought stress simulated by PEG 6000. Among the 1624 genes differentially expressed under drought, 97 were expressed in both genotypes. The transcription factors AP2-EREBP, bHLH, bZIP, C3H, MYB, NAC, WRKY, and MADS were involved in this mechanism. 13 *A. thaliana YUCCA6* ( *AtYUCCA6* ) mediates the biosynthesis pathway of tryptophan-dependent auxin in *A. thaliana* , which inhibits the formation of sweet potato storage root. Transgenic sweet potato plants expressing the *AtYUCCA6* gene under the regulation of SWPA2 promoter exhibited high-auxin phenotype, and *AtYUCCA6* -mediated auxin overproduction was associated with tolerance to oxidative and drought stresses. 14 Differential transcriptome analysis of drought-tolerant (C306) and drought-sensitive (WL711) genotype wheat showed significant induction or repression of genes involved in secondary metabolism, nucleic acid synthesis, protein synthesis, and transport in C306 compared with WL711. Significant upregulation and downregulation of transcripts of enzymes, hormone metabolism, and stress response pathways were observed in C306 under drought conditions. Other regulatory genes such as MT, FT, AP2, SKP1, ABA2, ARF6, WRKY6, AOS, and LOX2 were involved in the drought-resistant defense response of the C306 genotype. 15

### Low Temperature and High Temperature Stresses

Cassava ( *Manihot esculenta* ) can adapt to drought conditions, but it is sensitive to cold. The expression of *MeTCP4* , a transcription factor involved in plant development and abiotic stress, is altered under cold stress, and an increased expression of *MeTCP4* can enhance the plant ability to resist cold stress. The mRNA of *MeTCP4* -overexpressing plants and wild-type (WT) plants was isolated for whole genome sequencing, and the DEGs affected by *MeTCP4* overexpression under normal and cold conditions were identified as 1341 and 797, respectively. After cold treatment, some DEGs were found to be involved in the process of reactive oxygen species (ROS) metabolism. *MeTCP4* overexpression led to an increased expression of cold response genes and ROS clearance-related genes in plants, which may be the reason for the decrease in ROS levels and the enhancement of cold resistance in transgenic plants. 16 Under the condition of cold acclimation of *Eucalyptus globulus* plants, analysis of the leaf and root RNA-sequencing data identified a total of 51 *EglWRKY* genes, with an activation of the expression of multiple *EglWRKY* genes. There were 11 *EglWRKY* genes in the leaf tissues that were regulated during cold acclimation. 17 Comparative transcriptome analysis of anther development in rice in cold regions under cold stress demonstrated that starch metabolism and sucrose metabolism were the only important pathways (including 47 DEGs) that were annotated during the cold periods. 18 The basic helix-loop-helix (bHLH) transcription factors are involved in several abiotic stress responses. PEG and cold stress can induce 22 and 17 bHLH genes in the grape plant ( *V. vinifera* L.), respectively. PEG or cold treatment can significantly induce the VvbHLH007 (MYC2) gene. Six ICE genes were not upregulated. The transcription factors CBF, Myb, Hsf, and other bHLHs may regulate some members of the grape bHLH family. These transcription factors may be induced by abiotic stress and other transcription factors. 19 Several genes in *Dendranthema morifolium* encoding important transcription factors (CBF/DREB, bHLH, MYC, and ZAT) and proteins, and involved in cold signal transduction (CCX, CBP, CML, and MAPK), are upregulated or downregulated at low temperatures, and these genes may be involved in cold signal transduction. Several unigenes (20%) encoding unknown proteins were identified, and some unknown unigenes were found to be involved in the regulation of the response of *D. morifolium* to cold stress. 20 High-temperature stress altered the expression of a large number of transcripts of the Korean fir ( *Abies koreana* ). Under the combined action of high CO _2_ and high-temperature stresses, there was less alteration in gene expression changes, and important transcription factors such as ERF, bHLH, and NAC were identified. The differentially expressed transcripts are primarily related to light responses, biotic and abiotic stress responses, and development. 21 The response of the tea tree calcium-mediated transcription level to heat stress indicated that the CML gene may have a positive effect on heat stress response. This DEG was primarily related to signal transduction, transcription regulation, and posttranslational modification. The overexpression of the *CsCML45* gene significantly improved the survival rate and the heat tolerance of the heat-stressed transgenic *A. thaliana* . 22

### Salinity and Heavy Metal Stress

Cadmium (Cd) is a phytotoxic heavy metal pollutant, and 11,294 DEGs were significantly expressed in *Brassica juncea* under cadmium stress. Genes related to chemical stress, oxidative stress, and transport and secondary metabolic processes were upregulated. Cd-related transporters such as metal transporter (Nramp1), metal tolerance proteins (MTPC2 and MTP11), cadmium transporter ATPase, and plant cadmium resistance Sex proteins (PCR2 and PCR6) were all upregulated, whereas genes related to developmental processes and photosynthesis of multicellular organisms were downregulated. Cadmium/zinc transporter ATPase (HMA2, HMA3, and HMA4), high-affinity calcium reverse transport protein (CAX1), and iron transporter (IRT1) were downregulated. 23 Zinc oxide (ZnO) nanoparticles (nZnO) have harmful effects on plants. Transcriptome data show that both nZnO and Zn ^2+^ inhibit the growth of the plant's primary root (PR). nZnO inhibits the growth of PR through physical interaction and transcriptional regulation to destroy the tissue and structure of cell walls, whereas Zn ^2+^ has a greater cytotoxic effect on meristems. 24 The transcription factor MdMYB46 can enhance the tolerance of apples to salt and osmotic stress. MdMYB46 promotes secondary cell wall biosynthesis and lignin deposition by directly binding to the promoter of genes related to lignin biosynthesis, and it can also be activated directly by stress response signals to enhance the salt tolerance and osmotic stress tolerance of the apple plant. 25 Comparative salt transcriptome analysis of salt-sensitive and salt-tolerant maize inbred line (L87) showed that L87 has 1856 unique DEGs that were upregulated, including heat shock proteins (Hsp70s) and aquaporins in the 70-kDa family. The DEGs involved in the signal transduction of ABA, ETH, JA, and SA may affect the salt tolerance between the two varieties. In L87, SnRK2, the central component that positively regulates ABA signaling, was upregulated. DEGs related to active oxygen scavenging may enhance its salt tolerance, and WRKY TFs may cause salt tolerance differences between the two maize lines. 26 The response of the eggplant to salt stress is genotype-specific and organ-specific. Higher transcription levels of certain specific genes can improve salt tolerance, including C2C2-CO-like, WRKY, members of the MYB and NAC families. *AKT1* , *KAT1* , and *SOS1* were upregulated only in the leaves of salt-tolerant varieties. Heterologous expression of *SmAKT1* enhances the salt tolerance of yeast and *Arabidopsis* akt1 mutants. 27 Salt stress transcriptome analysis of *Salix* demonstrated that the upregulated genes were mainly involved in plant hormone pathways and β-alanine, galactose and betalain metabolism. ETH was found to be the primary signaling molecule that activated TF for 12 hours under salt stress. The upregulated genes were associated with elevated levels of amino acids, sucrose, inositol, stress proteins, and ROS scavenging enzymes, which help maintain water balance. 28 Overexpressing the *HvBADH1* transgene in transgenic wheat was found to significantly improve the overall salt tolerance of target plants and reduce the damage caused by high-salinity. 29 OsRH58 expression was upregulated in rice under salt, drought, or high-temperature stress, but it was decreased under low-temperature, UV, or ABA treatment. Under high-salt and drought stresses, the levels of several chloroplast proteins in the OsRH58-expressing *Arabidopsis* plants were elevated, indicating that OsRH58 transported by chloroplast exhibited RNA chaperone activity, which could enhance the stress tolerance of plants by increasing the translation of chloroplast mRNA. 30 Transcriptome analysis of *Oryza sativa* (rice) roots and stems under salt stress showed that salt stress significantly affected carbohydrate and amino acid metabolism and induced secondary metabolite-related genes, phenolic salts, and flavonoid accumulation in roots. 31 Transcriptome analysis of root toxicity stress in *A. thaliana* suggested that the sulfur metabolic pathway is activated only under severe aluminum stress, and cadmium and copper stresses triggered common transcriptional responses that may be related to the severity of stress. 32

### Waterlogging Stress

Under waterlogging stress, genes, which play a role in antioxidant systems, glycolysis and fermentation pathways, chlorophyll metabolism and amino acid metabolism, and certain hormones are activated. Many genes involved in chlorophyll synthesis and photosynthesis are downregulated, whereas may genes involved in stress tolerance are upregulated. In this way, the injury to grapes due to waterlogging can be reduced. ETH and ABA affect the response of plants to waterlogging, and alterations occur in the expression of genes associated with molecular functions (MFs) in the leaves. 33 Under salt and drought stresses, the expression of several stress-related genes in the transgenic lines was significantly downregulated, including those of endochitinase gene, peroxidase gene, potassium channel KAT3-like gene, and *SlMAPKKK11* gene. The *SlbZIP1* gene exerts salt and drought tolerance by regulating the ABA-mediated pathways. 34

## Outlook

Transcriptome technology can rapidly predict the relevant defense factors of stress and disclose the relationship between metabolic pathways, signal transduction, and defense response, which is of great significance in improving plant stress resistance and understanding the plant stress resistance mechanism. Plants respond to stress through a complex array of cellular, molecular, and physiological processes, and their metabolic networks are intricate. Although several genes related to the antistress metabolic pathway in plants have been cloned, and their molecular mechanism has been gradually elucidated, the understanding of plant antistress is still limited, and the synergistic effect of these pathways or other associations need to be further studied. When plants can tolerate various stresses in the natural environment, there may be a cross-interaction. Understanding the signal transduction and metabolic pathways that are specific and cross-shared by plants is a key topic in the study of plant resistance genes. In this process of research, we must use a variety of methods to comprehensively and accurately reflect the molecular mechanism of plant resistance.
